# Co-occurrence of feline chronic gingivostomatitis and oral squamous cell carcinoma in 4 cats (2014–2024)

**DOI:** 10.3389/fvets.2025.1564674

**Published:** 2025-04-29

**Authors:** Anson J. Tsugawa, Maria M. Soltero-Rivera, Stephanie Goldschmidt, Boaz Arzi, Tessa Kell, Naomi Hoyer, Cynthia M. Bell, Hanzhi Gao, Guogen Shan, Natalia Vapniarsky

**Affiliations:** ^1^William R. Pritchard Veterinary Medical Teaching Hospital, School of Veterinary Medicine, University of California, Davis, Davis, CA, United States; ^2^Department of Surgical and Radiological Sciences, School of Veterinary Medicine, University of California, Davis, Davis, CA, United States; ^3^Veterinary Institute for Regenerative Cures, School of Veterinary Medicine, University of California, Davis, Davis, CA, United States; ^4^Department of Clinical Sciences, College of Veterinary Medicine and Biological Sciences, Colorado State University, Fort Collins, CO, United States; ^5^Specialty Oral Pathology for Animals (SOPA), Geneseo, IL, United States; ^6^Department of Biostatistics, University of Florida, Gainesville, FL, United States; ^7^Department of Pathology, Microbiology, and Immunology, School of Veterinary Medicine, University of California, Davis, Davis, CA, United States

**Keywords:** FCGS, oral SCC, epithelial dysplasia, proliferative inflammation, cancer, neoplasia

## Abstract

**Introduction:**

Cats with refractory feline chronic gingivostomatitis (FCGS) exhibit chronic oral inflammation despite surgical and medical therapy. Such areas may resemble or be at higher risk for oral squamous cell carcinoma (SCC). Without routine biopsies, occult SCC may remain undiagnosed.

**Objectives:**

This study investigated the prevalence and potential association of oral SCC occurrence in cats with refractory FCGS.

**Methods:**

A retrospective review of cats with refractory FCGS and oral SCC from two veterinary teaching hospitals (2014–2024) was conducted. Cases with histopathologically confirmed FCGS, SCC, or both were included. Data analyzed included signalment, medical history, treatment, clinical findings, and diagnostics.

**Results:**

Two hundred twenty-one cats with refractory FCGS and 24 cats with oral SCC at the first institution, and 32 cats with refractory FCGS and 16 cats with oral SCC at the second institution, were presented over a 10-year period. Only four cats from both institutions had co-occurrence of FCGS and oral SCC. All affected cats exhibited bilateral proliferative FCGS lesions in the caudal oral cavity and developed SCC within 16–29 months (mean: 22 months). Two of four cats had epithelial dysplasia at FCGS diagnosis. SCC occurrence was significantly, inversely associated with FCGS at one institution (0.9%, *ρ* = −0.1424, *p*-value = 0.00035) but not the other (5.88%, *ρ* = 0.0495, *p*-value = 0.1947).

**Conclusion:**

Co-occurrence of FCGS and SCC is rare. While SCC may develop in proliferative FCGS areas, the low occurrence does not establish FCGS as a predisposing factor for SCC.

## Introduction

Feline chronic gingivostomatitis (FCGS) is an immune-mediated and inflammatory oral mucosal disease that may affect up to 26% of domestic cats and, more commonly, those living in multicat households (1–3). FCGS is a debilitating disease, and cats suffer from cripplingly painful oral mucosal lesions with clinical signs of ptyalism, partial to complete anorexia, reduced quality of life (poor grooming, reduced social interaction, lifelong medication administration), and in rare cases may be life-threatening (2–4). FCGS has an incompletely understood etiology characterized by an inappropriate immune response to co-infections of viruses such as feline calicivirus and puma feline foamy virus and an imbalance of the subgingival microbiome (1, 5–7).

There are three generally accepted phenotypic forms of FCGS: ulcerative, proliferative, and ulceroproliferative (3). Lesions may remain limited to one anatomic region of the mouth, mainly the alveolar/buccal mucosa, area lateral to the palatoglossal folds, or combined without clear demarcation lines with adjoining mucosal areas such as the peri-tonsillar mucosa and caudal sublingual mucosa. FCGS has been linked to semi-generalized or generalized, moderate or severe periodontitis, exhibiting a greater frequency of external inflammatory root resorption and retained roots compared to cats without FCGS (8). The proliferative and ulceroproliferative forms of FCGS may assume a neoplastic-like appearance and must be differentiated from other proliferative inflammatory lesions commonly seen in cats, such as eosinophilic granuloma complex and pyogenic granuloma (9, 10). While most FCGS cats present with bilateral mucosal lesions, unilateral manifestations also occur. Unilateral, particularly proliferative, presentations of FCGS are the most challenging to differentiate from neoplastic lesions such as oral squamous cell carcinoma (SCC) because of their similarities in clinical appearance. Concurrent inflammatory (and septic) or dysplastic changes in the examined histologic or cytologic samples may further blur the distinction between these entities. The finding of a more plasma cell-rich lymphoplasmacytic inflammatory infiltrate typically favors the diagnosis of FCGS, but an inflamed oral SCC can be difficult to rule out, especially if the evaluated samples are small and superficial or based only upon cytological evaluation (11). Biopsy locations caudal in the oral cavity may be difficult to access, hinder visibility, and make the procurement of representative tissue samples challenging, resulting in an inaccurate or incomplete diagnosis (12).

Achieving definitive resolution of FCGS clinical signs remains challenging, with as many as 30% of cats suffering from refractory disease following extraction therapy (13). Besides the suffering caused by chronically unresolved clinical signs, there may be significant local destructive effects to the tissues related to chronic inflammation, combined with alterations in the tissue microenvironment, cellularly and through cytokines, chronic inflammation which could influence the eventual transformation of refractory areas of inflammation into oral SCC (14). Aside from the informal discussion of this suggestion within professional circles, to the authors’ knowledge, there is no information in the literature regarding the confirmed prevalence or clinical features of co-occurrence of FCGS and oral SCC. The aim of this report was to identify and describe cases from two university teaching hospitals in which cats were affected by refractory proliferative FCGS and later diagnosed with oral SCC. We also aimed to examine the prevalence of co-occurrence of these conditions in the context of the general FCGS and oral SCC affected cat population from the two institutions.

## Materials and methods

### Case selection

A search of the electronic medical records databases of the William R. Pritchard Veterinary Medical Teaching Hospital, University of California-Davis (UCD VMTH), and James L. Voss Veterinary Teaching Hospital, Colorado State University (CSU VTH), was conducted for cats that were presented to the Dentistry and Oral Surgery Services (DOSS) of both institutions between 2014 to 2024 using keyword searches of cat, feline chronic gingivostomatitis (FCGS), FCGS, stomatitis, oral squamous cell carcinoma (SCC), and SCC. Cats with a diagnosis of FCGS or oral SCC or both were considered for inclusion, with the goal of identifying the prevalence of co-occurrence. Histopathological confirmation was required to be considered a confirmed case of oral SCC. And a confirmed case of FCGS required histopathological confirmation by mucosal biopsy, or cytological documentation if mucosal biopsy was never performed, and a history of typical clinical signs and/or ongoing therapy exceeding the accepted mean survival time (MST) of untreated oral SCC (3–4 months) (15), coupled with examination by a Board Certified Veterinary Dentist™. The tertiary referral case population of the veterinary teaching hospitals naturally selected for FCGS cats with refractory disease (i.e., absence of clinical response 6 months after treatment) (4). The medical records of cats that were identified with confirmed diagnoses were further evaluated for completeness, and only cats with complete records were included as cases studies.

### Medical records review

The following data were collected for each cat: (1) signalment, (2) medical history and treatment, (3) physical examination, (4) clinical laboratory findings, (5) histopathology and/or cytology, and (6) diagnostic imaging findings. Specific medical history details of interest included the course (months) of disease from initial diagnosis of FCGS to diagnosis with oral SCC, and description of other co-morbidities. FCGS treatment received was divided into three categories: surgical intervention (partial- and full-mouth tooth extractions, CO_2_ laser ablation), adjunctive medical therapy (antimicrobial, immunosuppressive, pain medications, NSAIDs, feline recombinant omega interferon), and mesenchymal stromal cell (MSC) therapy. Physical examination data included clinical phenotype of gingivostomatitis (ulcerative, proliferative, or ulceroproliferative), lesion’s location (buccal mucosa, alveolar mucosa, gingiva, molar salivary gland, lateral to palatoglossal folds, oropharyngeal, lingual or sublingual, nasal frenulum, lip), location of SCC and presence of lymphadenopathy. The clinical laboratory data that were evaluated included the CBC results (anemia, neutrophilia, or leukocytosis), specific serum chemistry parameters (total protein, globulin, and glucose), viral testing (FeLV/FIV), and, if available, upper respiratory panel (FHV-1, FCV, *Bordetella bronchiseptica*, *Chlamydophila felis*, *Mycoplasma felis*) and *Bartonella* blood test results. Biopsy reports were independently reviewed (NVA, CB) for confirmation of diagnosis (FCGS and/or SCC). Results of IHC testing, regional lymph node cytology, and necropsy findings, if available, were also reported. Diagnostic imaging review included all available modalities (intraoral radiographs, CT/CBCT, ultrasonography, echocardiography) to assess local, regional, and distant disease extent, evaluate for other sources of dentoalveolar inflammation/infection (e.g., periodontal or endodontal disease), and identify relevant comorbidities.

### Statistical analysis

Descriptive statistics were used to analyze the teaching hospital population data from 2014 to 2024 to determine disease rates of occurrence and prevalence of FCGS, oral SCC, and cats with FCGS and oral SCC. Fisher’s exact test was used to test the association of disease occurrence and prevalence as a comparison between hospitals (UCD VMTH and CSU VTH) and separately for FCGS, oral SCC and cats with FCGS and oral SCC. Occurrence describes how often a disease appears, while prevalence is an indication of how widespread the disease is at a specific point in time. Fisher’s exact test was used to compare the SCC occurrence rate in FCGS cats with the rate in cats without FCGS (16). All statistical analyses were performed using R for Windows version 4.2.3 (R Development Core Team, 2023, Vienna, Austria).

## Results

### Occurrence and prevalence of disease

The total number of cats, cats affected with feline chronic gingivostomatitis (FCGS), cats affected with oral squamous cell carcinoma (SCC), and cats with both FCGS and oral SCC seen between 2014 and 2024 by the Dentistry and Oral Surgery Services at both hospitals (UCD VMTH and CSU VTH) and their descriptive statistics are presented in Table 1. Since both institutions obtain mucosal biopsies of all FCGS cats that are presented, irrespective of phenotype, all FCGS cats identified in our search were confirmed cases with a pathological diagnosis with FCGS. The rates of FCGS occurrence and prevalence and oral SCC occurrence and prevalence were significantly (*p*-value <0.05) different between hospitals (Table 1). The occurrence and prevalence rates of cats with cooccurrence of FCGS and oral SCC were not significantly (*p*-value = 1) different between hospitals (Table 1). There was a significant association between FCGS and oral SCC at the UCD VMTH, with a lower percentage of cats with FCGS developing oral SCC (0.9%, 2 out of 223) compared to cats without FCGS (7%, 24 out of 343) (*ρ* = −0.1424, *p*-value = 0.00035). No significant association was found between FCGS and oral SCC at the CSU VTH (*ρ* = 0.0495, *p*-value = 0.1947) (Tables 2, 3). Collectively, four cats (two from each institution), were identified that developed oral SCC 16–29 months (mean: 22 months) after diagnosis of FCGS.

**Table 1:** FCGS and SCC cases by Dentistry and Oral Surgery Services (2014–2024).

Variable	UCD VMTH	CSU VTH	Total	*p*-value (Between-Hospital) (Fisher exact)
Total number of cats	566	749	1,315	
FCGS	221 (39.05%)	32 (4.27%)	253 (19.23%)	<0.0001
SCC	24 (4.24%)	16 (2.14%)	40 (3.04%)	0.034
FCGS + SCC	2 (0.35%)	2 (0.27%)	4 (0.30%)	1

**Table 2:** Contingency table for FCGS and SCC at UCD VMTH.

		SCC		*p*-value: comparing rate difference with Fisher exact test
		No	Yes	0.00035
FCGS	No	319 (93%)	24 (7%)	
	Yes	221 (99.1%)	2 (0.9%)	

**Table 3:** Contingency table for FCGS and SCC at CSU VTH.

		SCC		*p*-value: comparing rate difference with Fisher exact test
		No	Yes	0.1947
FCGS	No	699 (97.76%)	16 (2.24%)	
	Yes	32 (94.12%)	2 (5.88%)	

## Case reports

### Case 1

**Figure 1:**
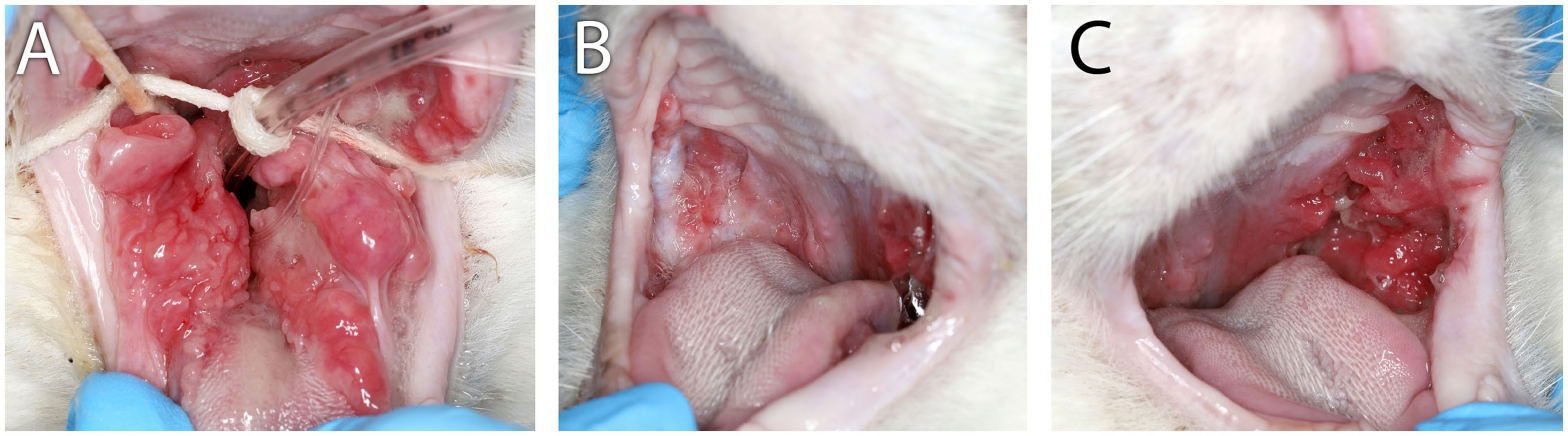
Clinical images of Case 1. **(A)** Clinical appearance of refractory proliferative phenotype bilateral caudal mucositis at 22 months following initial biopsy, full-mouth extractions, and CO_2_ laser ablation. **(B)** Marked reduction of inflammation intensity and proliferation of right-side caudal mucositis 3 months after receiving the second of two IV injections of 10 M uterine MSC that were administered 14 days apart. **(C)** Persistent left-side proliferative caudal mucositis following the second IV dose of uterine MSC.

**Figure 2:**
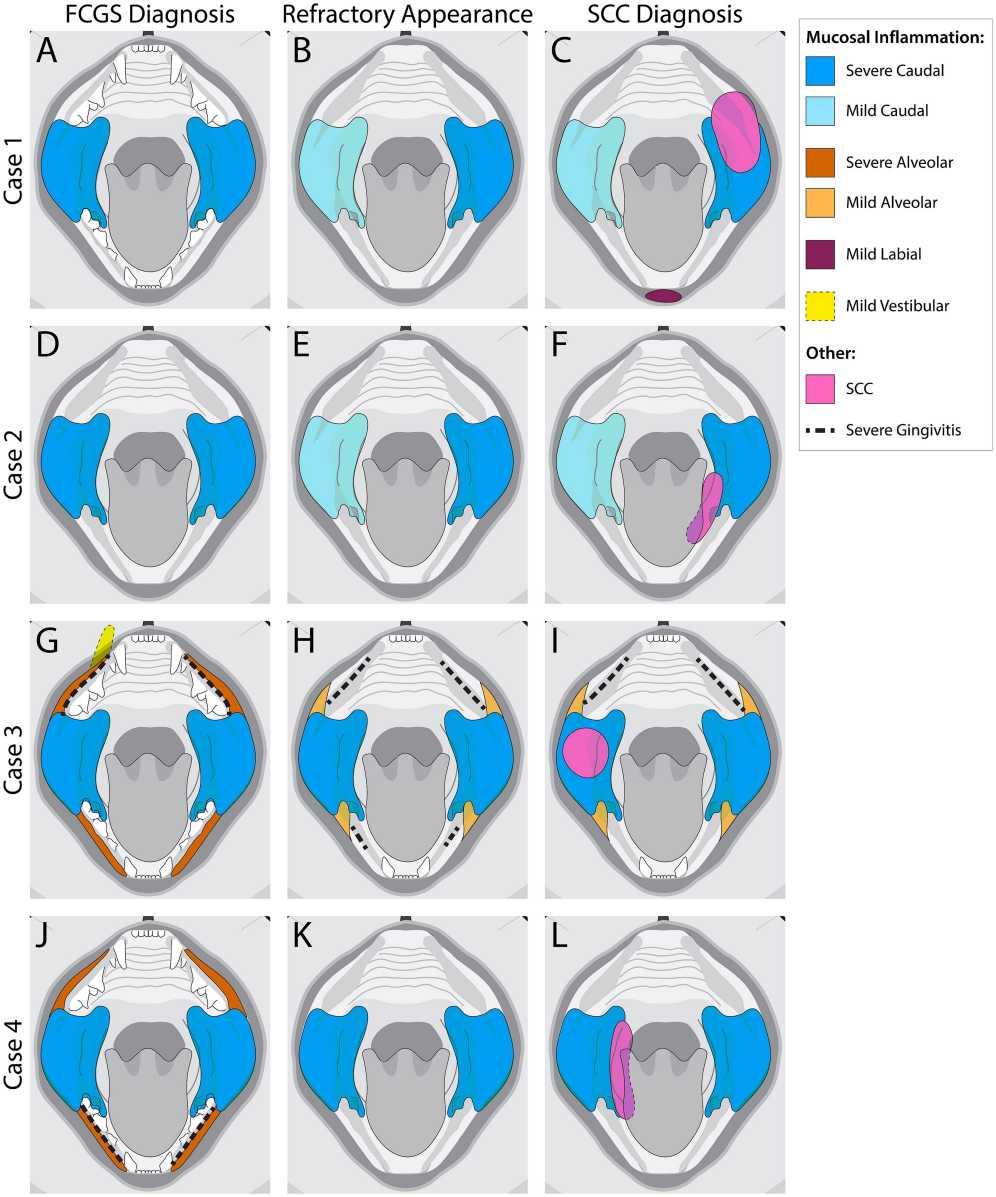
Visual representation of mucosal inflammation and oral squamous cell carcinoma (SCC) location in feline chronic gingivostomatitis (FCGS) cases at the time of FCGS Diagnosis, Refractory Appearance, and SCC Diagnosis. **(A–C)** Schematic representation of Case 1. **(D–F)** Schematic representation of Case 2. **(G–I)** Schematic representation of Case 3. **(J–L)** Schematic representation of Case 4. The colors represent affected areas and the severity of the inflammation noted at the different time points.

An indoor-dwelling 10-year-old neutered male Siamese cat weighing 7.4 kg (BCS 7/9) was presented for progressive anorexia and bloody ptyalism associated with long-standing (24 months), medically managed (Table 4) proliferative caudal FCGS (Figures 1A, 2A). The cat was cardiovascularly stable with a grade III/VI parasternal systolic murmur with likely primary hypertrophic cardiomyopathy confirmed by echocardiogram and IRIS Stage I borderline proteinuric chronic kidney disease. The remainder of the cat’s general physical examination and hematologic and clinical laboratory evaluations were unremarkable.

**Table 4:** Case demographics.

Case no	Age (yrs)	Sex	Breed	BW (kg)	Lifestyle	Duration of disease prior to presentation (mos)	Infectious	Clinical phenotype	Surgery	Adjunctive	Immunosuppressive	Immunomodulatory	FCGS Dx until SCC Dx (mos)	Comorbidities
1	10	MC	Siamese	7.40	indoors	24	FIV, FCV	proliferative	FME, CO_2_ laser	A, P, N	corticosteroids	MSC	20	Heart murmur (mild HCM); IRIS Stage I CKD
2	8	FS	DSH	7.00	indoors	60	FCV	proliferative	FME, CO_2_ laser	A, P	corticosteroids, cyclosporine	MSC	16	
3	7	MC	DSH	3.40	indoors	48	FHV	proliferative	PME	A, P, N	cyclosporine	rFeIFN-ω	25	
4	10	MC	Siamese	5.80	indoors	24	FIV	proliferative	FME, CO_2_ laser	A, P, N	cyclosporine	rFeIFN-ω	29	Physiologic heart murmur

FME, full-mouth extractions; PME, partial-mouth extractions; MSC, mesenchymal stromal cells; rFeIFN-ω, recombinant interferon omega of feline origin; A, antibiotics; P, pain medications; N, NSAIDs.

**Figure 3:**
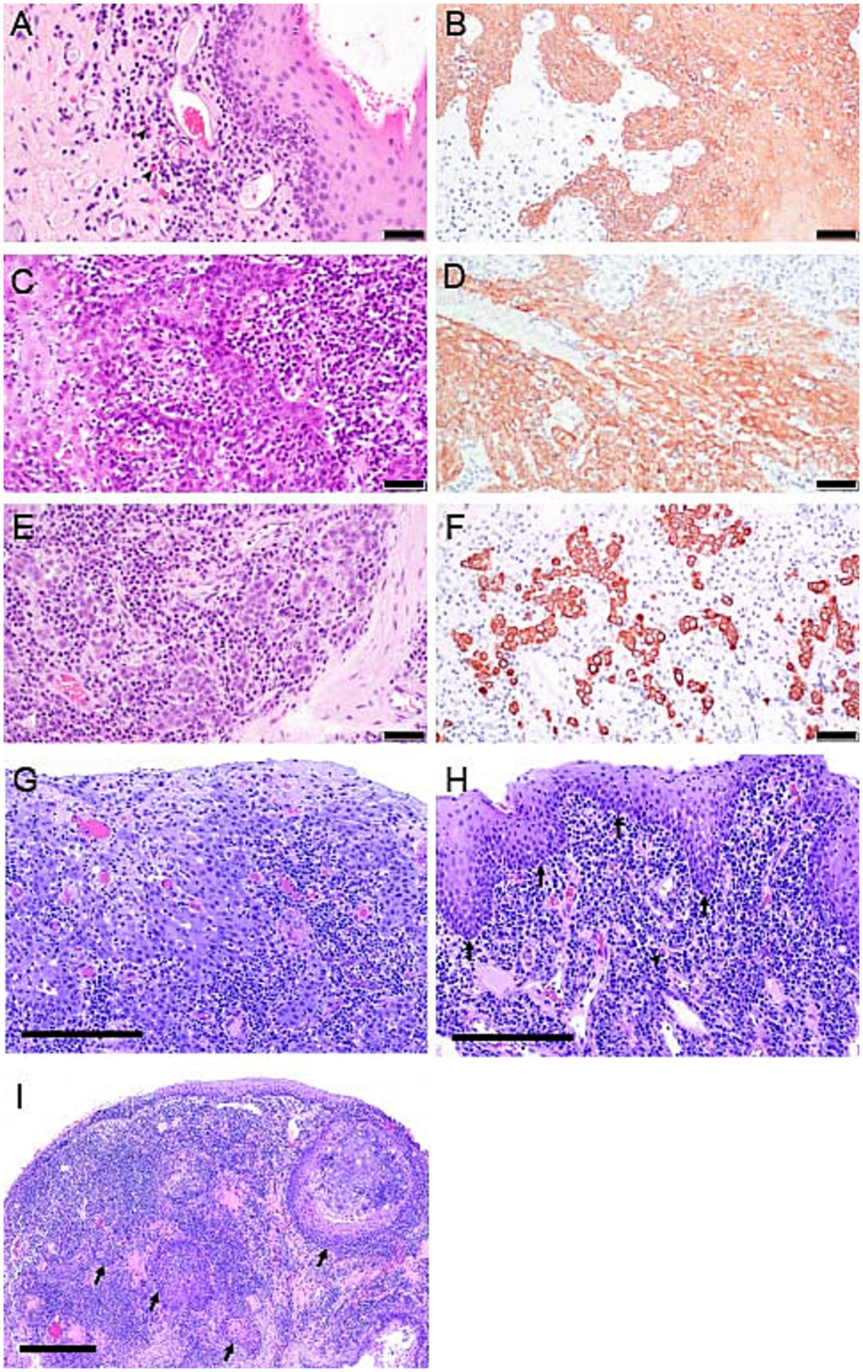
Photomicrographs from cases 1, 3 and 4. **(A)** Case 1, Photomicrograph of the palatoglossal fold with FCGS 20 months before diagnosis with SCC. Notice the infiltration of lymphocytes, plasma cells, and occasional plasma cells with Russell bodies (black arrowhead), also referred to as Mott cells. H&E stain, bar = 50 μm. **(B)** pan-CK immunolabeled section from case 1, 20 months prior to the diagnosis of oral SCC. Note marked epithelial hyperplasia with intradermal rete pegs. Pan-CK IHC, bar = 50 μm. **(C)** Case 1, 18 months before the diagnosis of oral SCC. Note marked epithelial hyperplasia and dysplasia with heavy lymphoplasmacytic infiltration of the lamina propria. Rete pegs are arborizing and heavily infiltrated with lymphocytes and plasma cells. H&E stain, bar = 50 μm. **(D)** pan-CK immunolabeled section of the proliferative oral mucosa from case 1, 18 months before the diagnosis of oral SCC. Dysplastic epithelium extends deep into the lamina propria, but still “respecting” the basement membrane. Pan-CK IHC, bar = 50 μm. **(E)** Case 1, at the time of diagnosis of SCC. Note individual epithelial cells and small cell clusters with no obvious basement membrane delineation extending deep into the lamina propria and surrounded by lymphoplasmacytic cellular infiltrate. H&E stain, bar = 50 μm. **(F)** pan-CK IHC photomicrograph from case 1, at the time of diagnosis of SCC. Notice the highlighted clusters of epithelial cells devoid of a basement membrane supporting the diagnosis of oral SCC. Pan-CK IHC, bar = 50 μm. **(G)** Case 3, photomicrograph of inflamed oral SCC from the biopsy of right caudal mucosa at the time of SCC diagnosis. Neoplastic squamous epithelial cells are relatively large with abundant, amphophilic (lavender) cytoplasm. Neoplastic cells lack orderly stratification along the mucosal surface and invade the lamina propria. Invading cells are associated with mixed inflammatory leukocytes. The majority of small, deeply basophilic (dark purple) cells are lymphocytes and plasma cells. H&E stain, bar = 200 μm. **(H)** Photomicrograph of proliferative, plasmacytic stomatitis from biopsies of caudal and sublingual mucosa from case 4 at CO_2_ laser ablation surgery 6 months before diagnosis of SCC. The surface mucosal epithelium is hyperplastic with extensions into the lamina propria, yet, regularly stratified and clearly delineated by the basement membrane (arrows). Fibrovascular tissue of the mucosal lamina propria is densely infiltrated by plasma cells, including Mott cells (arrowhead). H&E stain, bar = 400 μm. **(I)** Case 4, photomicrograph of oral SCC at the time of initial diagnosis, and six months following CO_2_ laser ablation. Note, multifocal islands of neoplastic squamous epithelium (arrows) are deep within the mucosal lamina propria, rimmed by plasma cells and lymphocytes. H&E stain, bar = 400 μm.

Anesthetized oral examination revealed severe, proliferative, caudally located mucositis and gingivitis involving the left sublingual surface and mucosa lateral to the palatoglossal folds, with the left side of the mouth more significantly affected than the right (Figure 2B). Full-mouth dental radiographs and dental charting revealed several missing teeth and generalized severe periodontal disease. The cat’s remaining teeth were extracted, and representative samples of the proliferative tissue were obtained for histopathologic evaluation. The location/side(s) of the mouth where the biopsies were obtained was not specified in the medical record. CO_2_ laser ablation was performed on the remaining proliferative tissue following the biopsy. The histopathologic evaluation described all sections of tissue as being heavily infiltrated by lymphocytes and plasma cells with severe epithelial dysplasia, acanthosis, ulceration, hemorrhage, edema, and granulation tissue (Figures 3A,B). The cat was discharged from the hospital with buprenorphine (0.01 mg/kg transmucosal twice daily) and gabapentin (5 mg/kg per os twice daily).

**Figure 4:**
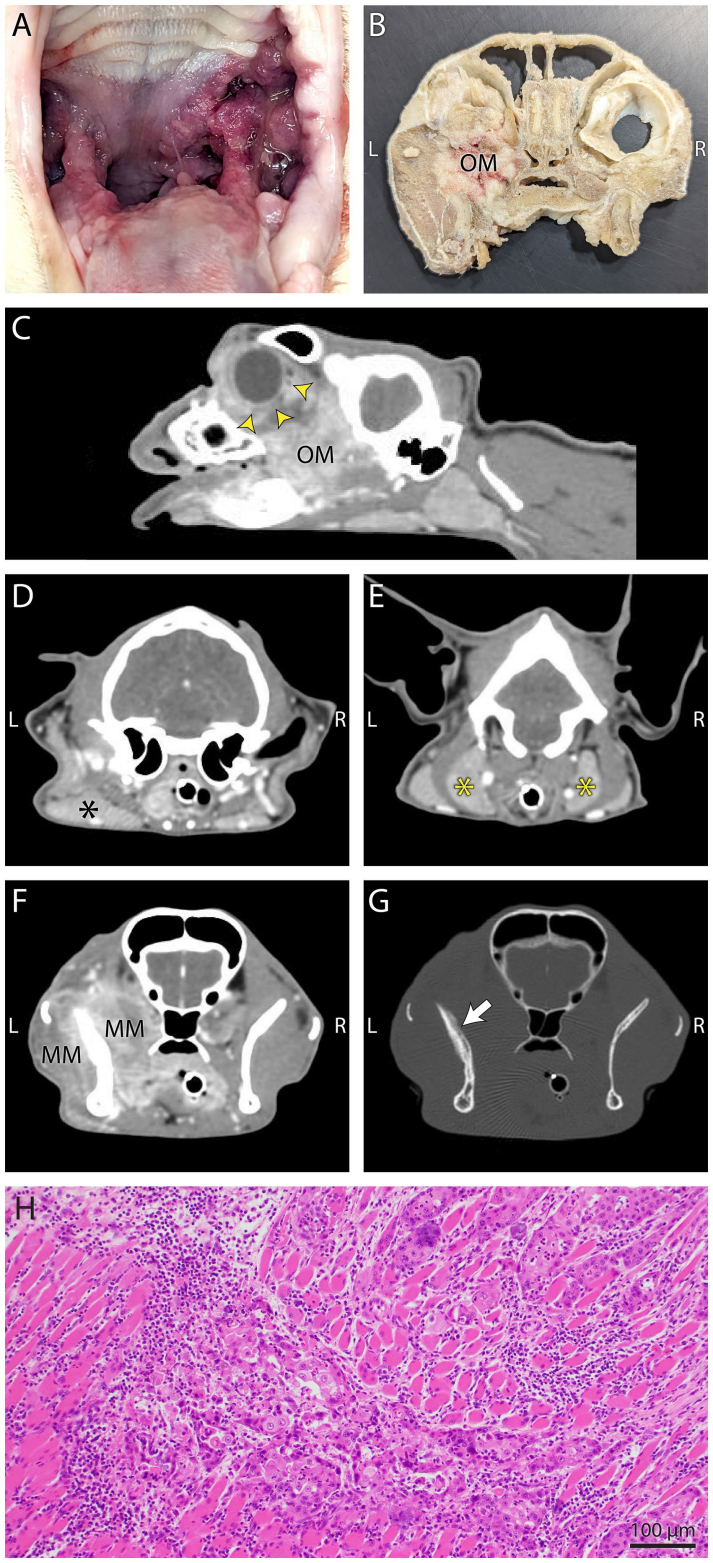
Gross necropsy pictures, computed tomography (CT) images, and histology from Case 1. **(A)** The mass on gross inspection was slightly firm, multifocal to coalescing, pink to red, multinodular and involved the left palatoglossal fold, periphery of the soft palate, buccal gingiva and caudal tongue. **(B)** Gross necropsy transverse section of the head with soft tissues removed. The infiltrative mottled pink to tan left maxillary mass (OM) has filled the OS retrobulbar space. **(C)** In this postcontrast sagittal plane reconstructed CT image the heterogeneously contrast enhancing oral mass (OM) has extended into the left retrobulbar space and has resulted in marked exophthalmos (yellow arrow heads). **(D,E)** In the left image, regional mandibular (black asterisk) lymphadenopathy, and in the right image, bilateral medial retropharyngeal (yellow asterisks) lymphadenopathy was evidenced on postcontrast transverse CT images. **(F,G)** Postcontrast CT images in soft tissue detail window setting (left) shows the mass infiltration of the masticatory musculature (MM), and the same image in a bone detail window setting (right), shows mildly irregular periosteal proliferation (white arrow) of the coronoid process of the left mandible. **(H)** Photomicrograph of a section from the skull of Case 1 with intravascular metastasis. Note the intramuscular invasion by neoplastic epithelial cells. H&E stain, bar = 100 μm.

The cat was presented for reevaluation at 2, 6, 10, 14, 19, and 35 weeks post extractions. There was progressive improvement in appetite and activity level for the first 6 weeks. However, ptyalism persisted, and although alveolar mucosal inflammation at the extraction sites improved, there was minimal improvement in caudal mucositis despite the extractions and adjunctive medical therapy that was offered (Table 4). At 18 months post-extraction therapy (Figures 3C,D), the cat received two intravenous injections of 20 million uterine MSCs 2 weeks apart. One month after receiving stromal cells, the cat’s activity level, grooming, and interest in food had improved, and oral examination revealed mild to moderate inflammation at the palatoglossal folds with a marked reduction in inflammation on the right side compared to the left side of the mouth (Figures 1B,C). Although the inflammation reduction on the right side of the mouth, attributed to the MSC therapy, was retained, 2 months later, the cat developed new clinical signs of head shaking, sneezing, and pawing at the mouth, and physical examination revealed decreased left ocular retropulsion with corneal erosion, pronounced ulceroproliferative mucositis lateral to and effacing the left palatoglossal fold, that extended to the lip, caudal left maxilla, left sublingual region, and floor of the mouth (Figure 2C). One of the two palpable left mandibular lymph nodes was firm and enlarged (30 mm). Thoracic radiographs revealed no evidence of pulmonary metastatic disease. A contrast-enhanced CT scan of the skull (Figures 4C–G) and thorax revealed an extensive heterogeneously contrast-enhancing left-sided maxillofacial mass with both pharyngeal and osseous involvement, left-sided exophthalmos, and suspected extension into the calvarium (Figure 4C). Significant regional lymphadenopathy (left lateral and medial mandibular lymph nodes, bilateral medial retropharyngeal) was evidenced with loss of distinction between nodes and heterogeneous contrast enhancement (Figures 4D,E). Cytological evaluation of fine-needle aspirates from the enlarged mandibular lymph nodes revealed moderate lymphoid and plasma cell hyperplasia without evidence of neoplasia. Histopathological evaluation of the proliferative oral mucosa from the caudal oropharyngeal region and lateral margin of the tongue base revealed ulcerated epithelium, lamina propria infiltration with lymphocytes, plasma cells, Mott cells, and rare eosinophils. Observation of areas of dysplastic and hyperplastic epithelium with islands of stratified squamous epithelial cells with keratinized cores extending into the lamina propria and not obeying the basement membrane created a concern for oral SCC (Figure 3E) which was further confirmed with pan-CK IHC (Figure 3F). Over the next several months the cat’s quality of life further declined, and humane euthanasia was elected by the owner. The cat’s necropsy revealed an infiltrative pink to tan mass (Figure 4A) that extended rostrally from the left maxilla to involve the left orbital bone, resulting in significant thinning of this bone, exophthalmos of the left eye (Figure 4B) and minimally involved the rostral portion of the left parietal bone. Bilateral cervical lymphadenopathy (1 × 1.5 cm and 1.5 × 2 cm) was observed. Histologic examination of the enlarged cervical lymph nodes revealed reactive hyperplasia, and no neoplastic cells were identified in the examined sections. Intravascular neoplastic cells were present in multiple regional blood and lymphatic vessels of the skull (Figure 4H).

### Case 2

An indoor-dwelling 7-year-old spayed female domestic shorthair cat weighing 7.00 kg (BCS 6/9) was presented for enrollment in an autologous adipose MSC clinical trial for non-responsive FCGS. The cat was clinically diagnosed with FCGS 60 months prior to presentation and had received staged surgical extractions over 33 months that concluded with the extraction of all teeth 24 months before trial enrollment. At presentation, the cat was reported to have a good appetite, was grooming and exhibiting good activity, but was experiencing oral pain and was receiving both cyclosporine and azithromycin for treatment of refractory FCGS. Other concurrent and previously prescribed therapies received over the prior 60 months, as well as the cat’s infectious disease status, are presented in Table 4.

**Figure 5:**
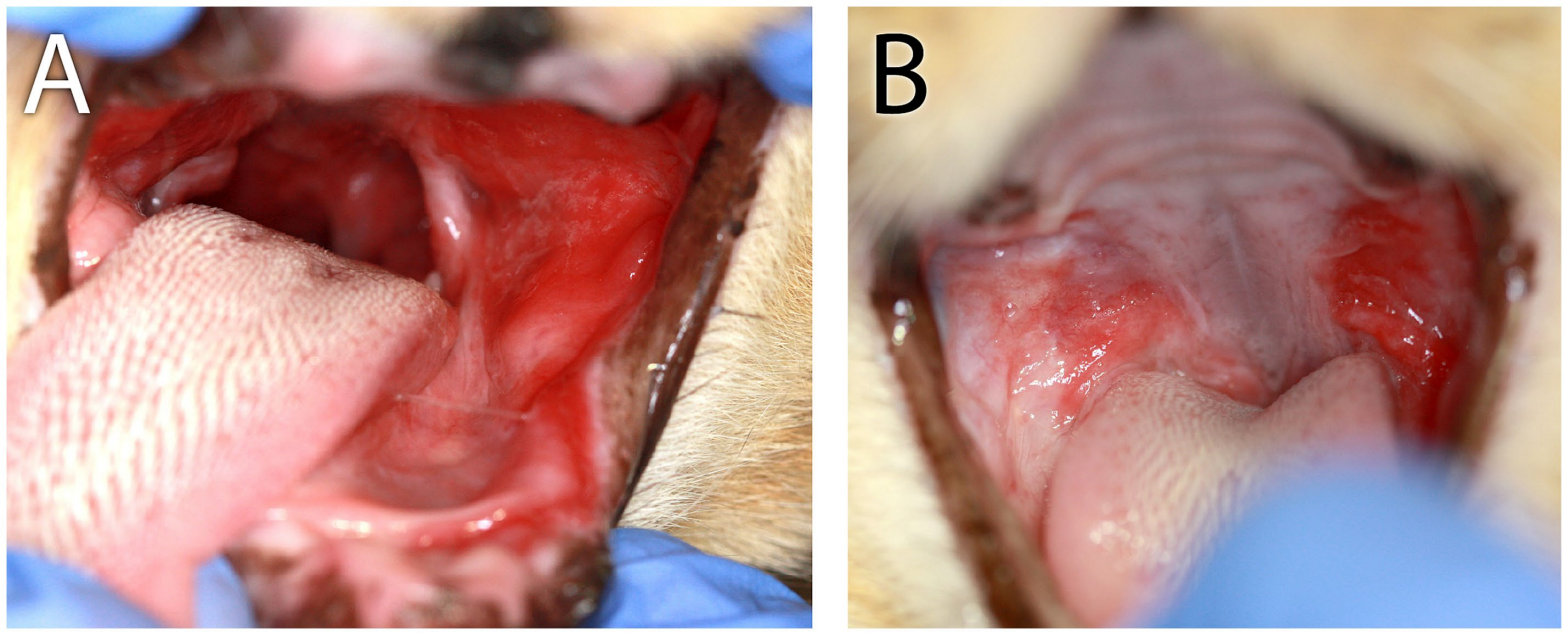
Clinical images of Case 2. **(A)** Initial refractory appearance of caudal mucositis. In this image, notice the severe mucositis lateral to and involving the left palatoglossal fold and sublingual region. **(B)** The appearance of caudal mucositis 1 month after receiving the second of two adipose MSC injections. Notice the clinical improvement of the right-side caudal mucositis.

No significant general physical examination abnormalities were detected. Oral examination revealed severe proliferative caudal mucositis with sublingual involvement (Figures 2D, 5A). Mandibular lymph nodes were enlarged bilaterally. Three unremarkable hematology and clinical chemistry tests were obtained between two to 4 months following enrollment into the clinical trial.

The cat underwent anesthesia for mucosal and mandibular lymph node biopsy and harvesting abdominal fat to isolate adipose stromal cells. Full-mouth dental radiographs confirmed the cat as edentulous. The cat was discharged from the hospital on buprenorphine (0.01 mg/kg transmucosal twice to three times daily for 2 weeks) and amoxicillin-clavulanic acid (13.75 mg/kg per os twice daily for 10 days). Concurrent prescriptions of cyclosporine and azithromycin were discontinued.

Mucosal and lymph nodes biopsies were evaluated by a board-certified anatomic veterinary pathologist (NVA) at the VMTH, and the reported pathological diagnoses were consistent with FCGS and moderate lymphoid and plasma cell hyperplasia.

The cat returned 2 weeks later for treatment with adipose mesenchymal stromal cell (20 million) injections that were administered intravenously 1 month apart, which were uneventful. Treatment with buprenorphine was continued.

Recheck examinations at 4 and 6 months after receiving the stromal cell therapy revealed an overall improvement in oropharyngeal mucositis on the right side of the mouth with persistence of proliferative tissue predominantly on the left side of the mouth (Figures 2E, 5B). The owner reported an improvement in appetite. The oral mucosal and incisional biopsy of enlarged mandibular lymph nodes were repeated upon exit from the trial at the 6-month follow-up examination. The reported post-trial pathological diagnoses of the mucosal and lymph node tissues were the same as pre-trial. The cat’s recovery from anesthesia was uneventful, and transmucosal buprenorphine (0.01 mg/kg) was continued post-operatively. A recheck in 12 months was recommended but not pursued. The cat was presented for recheck at 14 months, was no longer receiving buprenorphine, reported to be eating well, maintaining body weight, and oral examination revealed a similar pattern of inflammation to the 6 months visit with persistence of mucositis only on the left side of the mouth.

The cat was seen elsewhere 3 months later (16 months after receiving the second stromal cell injection) with a presenting complaint of oral bleeding. A caudal left sublingual mass was identified and biopsied (Figure 2F). Histopathological evaluation of the lingual tissue confirmed sublingual SCC with multifocal mild tubular or pseudo-glandular change. Twelve mitotic figures were encountered in 10 high-power fields. The poor prognosis associated with this diagnosis was discussed with the owner, and due to rapid enlargement of the tumor, tongue displacement, and inability to close the mouth, per the request of the owner, the cat was humanely euthanized 2 months following the oral SCC diagnosis.

### Case 3

An indoor-dwelling 7-year-old castrated male domestic shorthair cat weighing 3.43 kg (BCS 8/9) was presented for oral pain (difficulty eating and blood-tinged saliva) associated with an acute increase in the severity of clinical signs from refractory FCGS. The cat had been diagnosed with FCGS 48 months before presentation. The mucositis at diagnosis was described as severe alveolar and caudal with focal vestibular involvement at the level of the right maxillary canine tooth (Figure 2G). An initial histopathology report showed stomatitis, erosive to ulcerative, plasmacytic, diffuse, chronic, marked with granulation tissue and edema, consistent with FCGS. Mott cells, a characteristic cell found in the oral mucosa of cats with FCGS (3), were mentioned in this report. The exact location where the incisional biopsy samples were obtained was not described in the medical record, but multiple samples were evaluated, and the location of the inflammation was reported as caudal, sublingual, and in the right maxillary region. A Grocott–Gömöri’s methenamine silver histochemical stain for the identification of fungal organisms was negative. The cat was treated with caudal mouth extractions. A review of the dental radiographs obtained following the extractions confirmed the complete extraction of the premolar, molar, and maxillary canine teeth. Over the course of 2 years the alveolar mucositis lessened in intensity in response to the extractions, but the caudal mucositis remained stagnant (Figure 2H).

**Figure 6:**
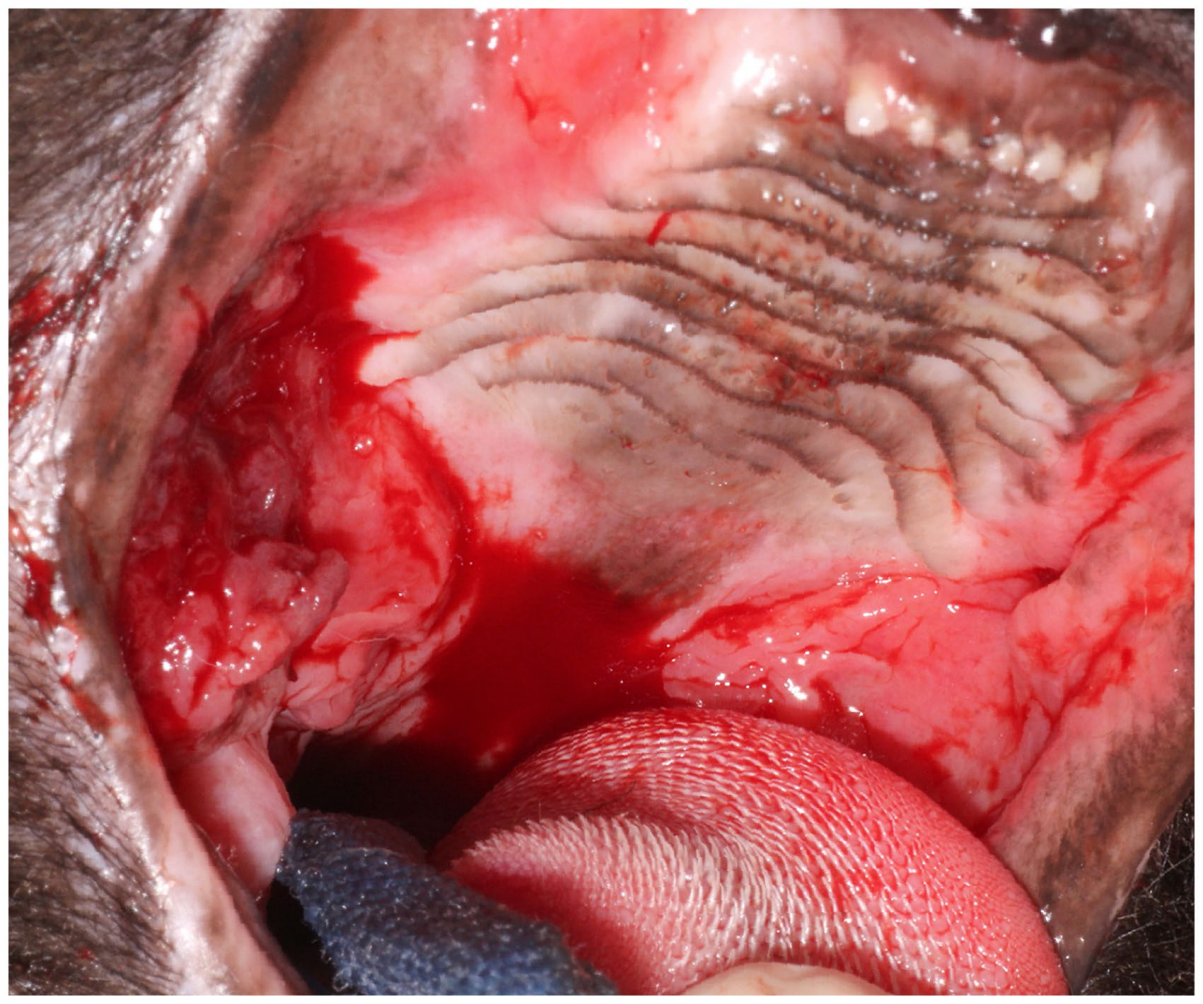
Clinical image of Case 3. Notice the severe ulcerative alveolar mucositis present at the level of the previously extracted maxillary teeth consistent with refractory disease of nearly 2 years following partial-mouth extractions. Areas of more proliferative caudal mucositis are present bilaterally and extend from the caudal hard palate to the tonsils. The dark pink-red, friable ulcerative multinodular oral mass that was later confirmed histologically as oral SCC was observed caudal to the hard palate and lateral to the right palatoglossal fold within the region of chronic proliferative mucosal inflammation.

At presentation, the cat was receiving gabapentin (5 mg/kg per os twice daily) and recently had received robenacoxib, cefovecin, and tramadol. Further details of the treatment received are summarized in Table 4. The cat’s hematologic and clinical chemistry tests were unremarkable, and viral (FeLV/FIV) tests were negative except for feline herpesvirus type-1. The general physical examination was unremarkable. Maxillofacial examination revealed swelling of the right side of the cat’s face, decreased retropulsion and epiphora of the right eye. There was a 34 × 52 mm ulcerated, friable mass in the right peritonsillar region that effaced the right palatoglossal fold and extended 5 mm into the hard palate with oral hemorrhage (Figures 2I, 6).

The cat was anesthetized for biopsy of the oral mass (2 years after the initial biopsy of FCGS). Histopathological evaluation of the oral mass confirmed the diagnosis of oral SCC of the right maxilla with severe plasmacytic/mixed inflammation (Figure 3G). Further follow-up for this cat was not available.

### Case 4

An indoor-dwelling 10-year-old castrated male Siamese cat weighing 5.8 kg (BCS of 7/9) with refractory proliferative phenotype FCGS was presented for CO_2_ laser ablation after having received full-mouth extractions and adjunctive medical therapies are summarized in Table 4. The cat was diagnosed with FCGS 24 months prior to presentation. The FCGS-associated oral inflammation at the time of diagnosis was severe bilateral alveolar and caudal mucositis with severe gingivitis limited to the mandible (Figure 2J). Following extractions the alveolar mucositis and gingivitis resolved, but the severity of caudal mucositis remained unchanged (Figure 2K).

On presentation the cat’s physical examination revealed enlarged mandibular lymph nodes (R > L), and an intermittent grade II/VI systolic parasternal murmur. The remainder of the general physical examination was unremarkable. Two sets of hematology and clinical chemistry tests separated by 5 months were available for review and were unremarkable. The cat was FIV positive. Clinical information on this patient is summarized in Table 4.

**Figure 7:**
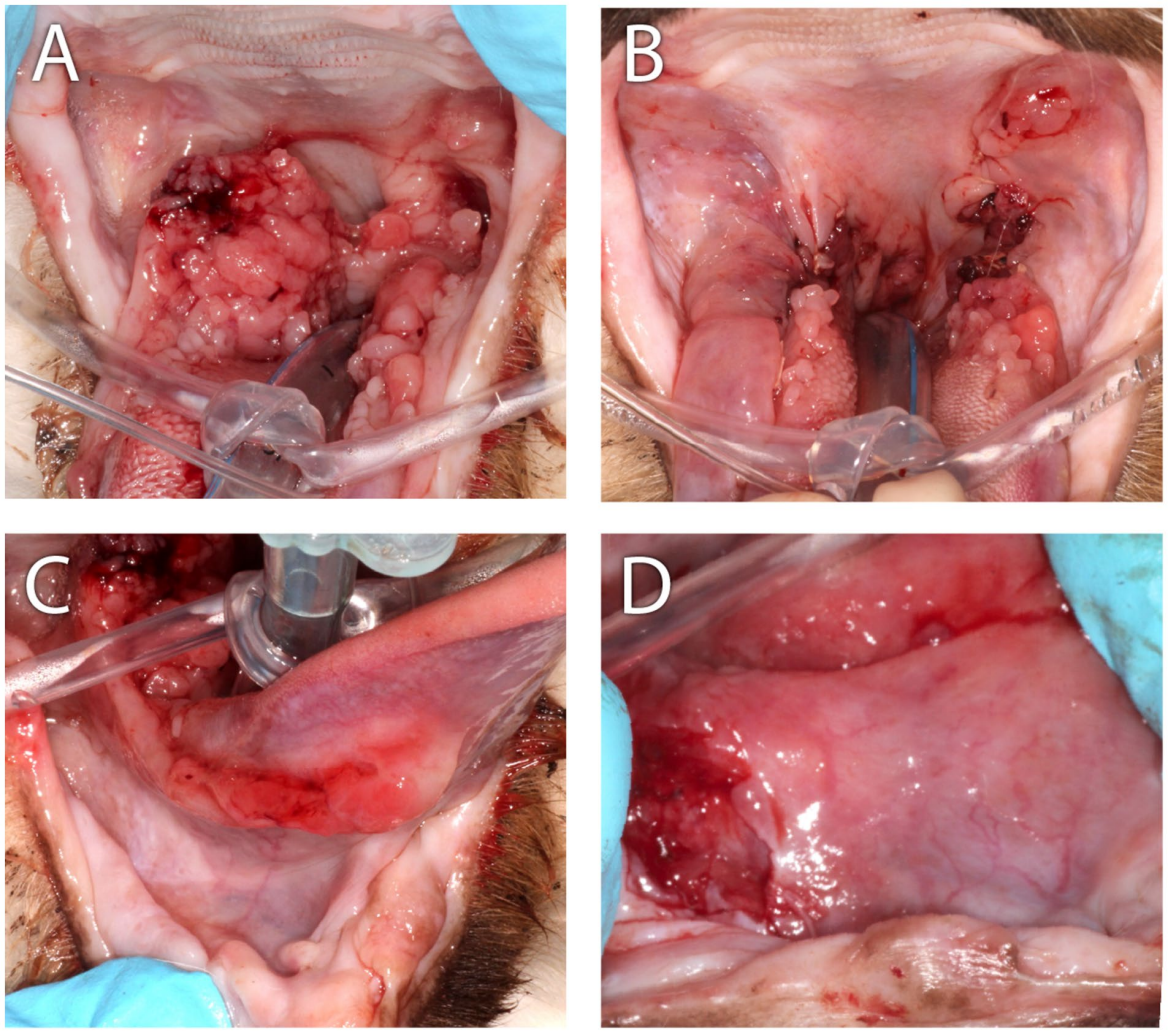
Clinical images of Case 4. **(A)** This case was presented with severe bilateral proliferative mucositis along the palatoglossal folds with infolding of bulky tissue into the airway that resulted in intermittent airway obstruction and clinical signs of snoring and apnea when sleeping. **(B)** Appearance of the caudal oropharynx immediately following CO_2_ laser ablation combined with scalpel debulking of the proliferative tissue. The grossly normal mucosal tissue was sutured over the ablated tissue. **(C)** At initial presentation, an ulcerative mucositis was observed along the right sublingual surface of the tongue. **(D)** Nearly 6 months following CO_2_ laser ablation, scalpel debulking of the proliferative mucosa, the cat was presented with a left-sided deviation of the tongue. In this close-up and cropped image of the sublingual surface, from the perspective of the tongue pulled rostrally and to the cat’s left, the ulcerated right sublingual mass was revealed.

Oral examination revealed multifocal mass-like proliferations of the oral mucosa lateral to and involving the palatoglossal folds (Figure 7A). The right-side proliferative lesion measured 25 × 18 mm and nearly occluded the airway. An additional 30 × 9 mm mass was present along the caudoventral aspect of the tongue on the right and 15 × 3 mm on the left. An ulcerative mucositis was observed sublingually along the right (Figure 7C). The lip commissures were ulcerated. Approximately 60–80% of the proliferative tissue was ablated with a carbon dioxide laser, and the adjacent healthy oral mucosa was used to cover the ablated tissue and sutured (Figure 7B). An esophageal feeding tube was placed. Over 20 multinodular soft tan/hemorrhagic incisional tissue samples obtained from the right palatoglossal fold and adjacent sublingual region were evaluated histologically, and were described as proliferative, erosive, and plasmacytic mucositis. All samples had similar histomorphology (Figure 3H). Anesthetic recovery was uneventful, and the cat was discharged home with esophageal feeding recommendations to meet the cat’s resting energy requirement, transmucosal buprenorphine (0.01 mg/kg once twice to three times daily) for pain, and amoxicillin/clavulanic acid (13.75 mg/kg twice daily), metoclopramide (0.5 mg/kg 30 min prior to esophageal tube feeding) and maropitant (2 mg/kg once daily) through the feeding tube.

For 2 months following CO_2_ laser ablation the cat experienced significant improvement in clinical signs that included a reduction in ptyalism and improved consistency in grooming and appetite. Upon return of the clinical signs of ptyalism, conscious oral examination revealed a mild proliferation of the glossopharyngeal tissue, and cyclosporine (2.5 mg/kg per os twice daily) was prescribed. Improvement of symptoms was not observed over 2 months while receiving cyclosporine therapy. A follow-up physical examination revealed weight loss of 0.25 kg, blood-tinged saliva, a moderate progression of the proliferative caudal mucositis, and a mass-like lesion on the ventral right tongue base that resulted in deviation of the tongue to the left side of the mouth (Figures 2L, 7D). Cyclosporine therapy was discontinued, gabapentin (50 mg per os twice to three times daily) was initiated for pain, and the cat was re-anesthetized for an incisional biopsy of this newly identified sublingual mass. The caudal right sublingual mass, measuring 10 × 20 mm, was noted to be ulcerated and friable. Three incisional biopsy samples were obtained and submitted for histopathologic evaluation. The cat recovered uneventfully from anesthesia and was discharged with gabapentin (5 mg/kg per os twice to three times daily) and prednisolone (1 mg/kg per os once daily). The samples were evaluated by the same pathologist (CMB) who reviewed initial biopsies 5 months prior. All samples were reported as oral SCC with 14 mitotic figures per 10 high-power field (Figure 3I). Following disclosure of the histopathologic diagnosis and associated poor prognosis with the owner, the cat was lost to follow-up.

## Discussion

The purpose of this case series was to examine the co-occurrence of feline chronic gingivostomatitis (FCGS) and oral squamous cell carcinoma (SCC) across a 10-year period at two institutions. During the decade of study, a significantly larger number of feline cases with the confirmed diagnosis of FCGS were identified in the search of the electronic medical records from the William R. Pritchard Veterinary Medical Teaching Hospital, University of California Davis (UCD VMTH) compared to the James L. Voss Veterinary Teaching Hospital, Colorado State University (CSU VTH), but a similar number of oral SCC cases were seen. Routine mucosal biopsy of all cats with FCGS was the protocol at both institutions, and therefore all FCGS cats were confirmed cases of FCGS with a pathological diagnosis of FCGS. We found that the co-occurrence of FCGS and oral SCC was very rare (4 cases over a 10-year period). All cases of FCGS and oral SCC co-occurrence had long-standing proliferative FCGS, and oral SCC developed within these refractory areas. Epithelial dysplasia was an early finding in proliferative caudal mucositis lesions in 2 of the cases that developed oral SCC.

Interestingly, despite documentation of co-occurrence of FCGS and oral SCC, at the UCD VMTH, a significant inverse association was identified between FCGS and oral SCC, meaning the presence of FCGS appeared protective for the development of oral SCC. This inverse association was not found at the CSU VTH. The latter could potentially be associated with differences in case management between the two hospitals, or statistical artifact, rather than a true protective effect from FCGS itself, and even explained by the greater variation resulting from the small sample size of FCGS cats seen at the CSU VTH. It should be noted, however, despite the smaller sample size of FCGS cases at the CSU VTH, all cases of FCGS were confirmed, as they were at UCD VMTH, with a pathological diagnosis of FCGS. The number of FCGS cases seen were quantitatively different between hospitals, with the UCD VMTH having seen a numerically larger number of FCGS cases than the CSU VTH, whereas the oral SCC caseload was similar between hospitals, and these differences were supported statistically (Table 1). The larger overall and refractory FCGS caseload seen at the UCD VMTH may be attributable to the availability of MSCs to control inflammation in refractory FCGS cats, which is unique to this institution (17–19). The actual number of refractory FCGS cats (or FCGS cats differentiated by phenotype) seen at each hospital was not able to be determined using the keyword searches that were performed. Phenotyping (2) and the refractory (4) classification of FCGS cats are newer nomenclature, inconsistently used in the medical records during this time period, and further designation of FCGS cats was not possible. The descriptive statistics applied to the teaching hospital caseload of both diseases separately and together parallels the quantitative data, and cats with co-occurrence of FCGS and oral SCC were presented very rarely over the decade of study. In concert, FCGS does not appear to predispose cats to the development of oral SCC.

The course of oral mucosal inflammatory disease in the four cats in this series with proliferative mucositis was chronic, with a mean of 39 months prior to presentation to the teaching hospital. The potentially deleterious effects of chronic inflammation are believed to occur on a molecular level, where chronic unresolved inflammation may activate pro-oncogenes, inactivate suppressor genes, and result in genomic instability and cell mutations that increase the risk of cancer (20, 21). The influx of leukocytes associated with chronic inflammation may also serve as an enriching source of tumor-promoting cytokines and growth factors (22). Although all cats with FCGS, regardless of phenotype, suffer from chronic inflammation, refractory cases have a longer course of inflammation, and with the added physical disguise of proliferative lesions, we suspected that the proliferative FCGS phenotype would be a risk factor for delayed or misdiagnosis of more sinister disease. Yet, the overall prevalence of disease co-occurrence was very rare, and at one institution was even possibly protective, questioning the significance of chronic inflammation in these proliferative areas of mucositis and cancer risk. However, based upon similarities in clinical appearance of proliferative FCGS lesions and oral SCC, as well as the severe consequences of misdiagnosis or delays in diagnosis may have on prognosis, routine histopathologic evaluation of areas of proliferative mucositis in FCGS cats is warranted for proper clinical care.

The four FCGS cats in our report all exhibited the proliferative (to ulceroproliferative) phenotype, had undergone surgical extraction as well as medical management, and remained partially to completely refractory for the duration of treatment. Treatment options for refractory patients are limited, and all cats in this series had received treatment with an alternative immunosuppressant agent (cyclosporine) to traditional corticosteroids and immunomodulatory therapy (recombinant feline omega interferon) and/or MSC therapy. Immunosuppressive therapies have significant side-effects and may even increase the risk of cancer development or progression through systemic immunosuppression and decreased tumor surveillance (23, 24). Human and feline organ transplant patients often receive cyclosporine for years, sometimes lifelong, and this lengthy exposure may predispose these patients to a six-fold (25) increased risk of skin SCC development, and in cats, a six-times higher odds of developing malignant neoplasia (24). Cyclosporine is used off-label for the treatment of refractory FCGS (26), and was used in three of the cases reported here, however, the treatment duration was intermittent or very short (2 to 6 months), and the authors do not attribute the development of oral SCC in these cats to cyclosporine due the brevity of exposure to the drug. At the time of oral SCC diagnosis, all cats had experienced a partial improvement in clinical signs (i.e., ptyalism, appetite, grooming and social interaction) and overall intensity of mucositis, predominantly observed as a reduction in alveolar mucositis. The therapeutic responses of cases 1 and 2 to MSC administration were especially noteworthy and correlated well with previous studies (3, 17–19). Despite the development of oral SCC on the contralateral (left) maxilla in case 1 and sublingual in case 2, the clinical reduction of unilateral caudal inflammation in the opposite side of the mouth remained through the eventual diagnosis of oral SCC (Figures 1B,C, 5B); the significance of this occurrence is unknown.

Epithelial dysplasia (ED) of proliferative mucositis was identified as an early cytological and histological finding in 2 cases reported in this series. This observation suggests a cellular predisposition to malignancy; however, it is not considered a precursor lesion of oral SCC as described in humans (27). ED in humans is a clinical disease entity characterized by cellular atypia within the squamous epithelial layer of the oral mucosa (28). Universally, dysplastic changes are not exclusive to carcinogenesis, and increased mitotic activity may also be identified in the reactive epithelium (20). Misdiagnosis of the initial FCGS diagnosis in these cats was considered, however, all cats in our series survived a median of 669 days (22 months) before diagnosis with oral SCC. Thus, misdiagnosis is less likely based upon this lengthy time frame, specifically for case 1 where the pan-CK pattern was not supportive of oral SCC on the initial biopsy specimen (Figures 3A–D). When dysplasia is identified histologically in proliferative FCGS lesions, these patients should be responsibly monitored and/or subjected to serial tissue biopsy with histopathologic evaluation over many months.

Due to the retrospective nature of this study, case omissions of co-occurrence may have occurred related to the inclusion criteria and search methodology used. Specifically, this case series focused on the refractory form of FCGS, and cats that responded favorably to extraction therapy and also developed oral SCC that may not have returned for follow-up with the same teaching hospital service would not have been recorded as cases of co-occurrence. The clinical severity of both diseases is presumed to have contributed to the majority of cases lost to follow-up. Electronic medical record searches may have missed cats that were presented to clinical services of the hospital less familiar in the clinical recognition of either disease, and FCGS and/or oral SCC may not have been recorded as diagnoses, and euthanized without biopsy. Considering the importance of our conclusions, with deference to the low case numbers of confirmed co-occurrence of FCGS and oral SCC encountered in the series, prospective longitudinal investigative studies are needed to further validate our findings and confirm the low cooccurrence rate of these diseases. Histological studies that expand upon the characterization of the inflammatory infiltrate (e.g., eosinophils) in refractory FCGS lesions (and oral SCC) and the significance of dysplastic epithelial changes may provide valuable prognostic information for FCGS cats (29, 30). Gene expression and molecular studies to better characterize dysplastic changes and clarify the role viral mutagenesis and chronic inflammation may have upon the transformative potential of these lesions would be a valuable continuation of this study (31–33). In consideration of the lasting inflammation reduction achieved through the immunomodulatory effect of MSCs in two of the cases presented here and previously (3, 17–19), especially as this therapeutic option becomes more readily accessible and available for introduction earlier in the course of disease, the mechanistic role of MSCs within the tumor microenvironment (34) should justly receive further investigation. The inflammation reduction of MSC therapy in these two cats was partial but strikingly unilateral (Figures 1B,C, 5B), which made the contralateral non-responsive areas of inflammation, where the SCC lesions eventually developed, visibly standout. The significance of this occurrence if unknown; however, it does introduce the question whether areas of persistent inflammation that do not improve following MSC therapy should receive further monitoring consideration due to the importance early diagnosis of oral SCC has upon treatment outcome.

## Conclusion

Co-occurrence of feline chronic gingivostomatitis (FCGS) and oral squamous cell carcinoma (SCC) is rare and, in the present study, a significant inverse association between the two diseases was identified. All cases of co-occurrence had a long-standing proliferative phenotype of FCGS. Despite the low occurrence, the results of this case series highlight the importance of obtaining baseline oral mucosal biopsies in proliferative phenotype FCGD, particularly when asymmetry is observed, due to the high morbidity and mortality caused by oral SCC. The careful observation of proliferative FCGS lesions over many months, especially areas of inflammation that remain refractory to treatment, the practice of obtaining serial biopsies of clinically and biologically suspicious areas, and utilization of IHC testing when indicated, may avoid misdiagnosis and/or significant delays in oral SCC diagnosis in cats.

## Data Availability

The original contributions presented in the study are included in the article/supplementary material, further inquiries can be directed to the corresponding author/s.
